# Hand Resting Tremor Assessment of Healthy and Patients With Parkinson’s Disease: An Exploratory Machine Learning Study

**DOI:** 10.3389/fbioe.2020.00778

**Published:** 2020-07-14

**Authors:** Ana Camila Alves de Araújo, Enzo Gabriel da Rocha Santos, Karina Santos Guedes de Sá, Viviane Kharine Teixeira Furtado, Felipe Augusto Santos, Ramon Costa de Lima, Lane Viana Krejcová, Bruno Lopes Santos-Lobato, Gustavo Henrique Lima Pinto, André dos Santos Cabral, Anderson Belgamo, Bianca Callegari, Ana Francisca Rozin Kleiner, Anselmo de Athayde Costa e Silva, Givago da Silva Souza

**Affiliations:** ^1^Núcleo de Teoria e Pesquisa do Comportamento, Universidade Federal do Pará, Belém, Brazil; ^2^Instituto de Ciências Exatas e Naturais, Universidade Federal do Pará, Belém, Brazil; ^3^Instituto de Ciências da Saúde, Universidade Federal do Pará, Belém, Brazil; ^4^Núcleo de Medicina Tropical, Universidade Federal do Pará, Belém, Brazil; ^5^Instituto de Ciências Biológicas, Universidade Federal do Pará, Belém, Brazil; ^6^Instituto de Ciências da Arte, Universidade Federal do Pará, Belém, Brazil; ^7^Centro de Ciências Biológicas e da Saúde, Universidade do Estado do Pará, Belém, Brazil; ^8^Departamento de Ciência da Computação, Instituto Federal de São Paulo, Piracicaba, Brazil; ^9^Laboratório Rainha Sílvia de Análise do Movimento, Rio Claro, Brazil; ^10^Departamento de Fisioterapia, Universidade Federal de São Carlos, São Carlos, Brazil

**Keywords:** Parkinson’s disease, inertial sensors, accelerometer, gyroscope, hand resting tremor, machine learning

## Abstract

The aim of this study is comparing the accuracies of machine learning algorithms to classify data concerning healthy subjects and patients with Parkinson’s Disease (PD), toward different time window lengths and a number of features. Thirty-two healthy subjects and eighteen patients with PD took part on this study. The study obtained inertial recordings by using an accelerometer and a gyroscope assessing both hands of the subjects during hand resting state. We extracted time and temporal frequency domain features to feed seven machine learning algorithms: k-nearest-neighbors (*k*NN); logistic regression; support vector classifier (SVC); linear discriminant analysis; random forest; decision tree; and gaussian Naïve Bayes. The accuracy of the classifiers was compared using different numbers of extracted features (i.e., 272, 190, 136, 82, and 27) from different time window lengths (i.e., 1, 5, 10, and 15 s). The inertial recordings were characterized by oscillatory waveforms that, especially in patients with PD, peaked in a frequency range between 3 and 8 Hz. Outcomes showed that the most important features were the mean frequency, linear prediction coefficients, power ratio, power density skew, and kurtosis. We observed that accuracies calculated in the testing phase were higher than in the training phase. Comparing the testing accuracies, we found significant interactions among time window length and the type of classifier (*p* < 0.05). The study found significant effects on estimated accuracies, according to their type of algorithm, time window length, and their interaction. *k*NN presented the highest accuracy, while SVC showed the worst results. *k*NN feeding by features extracted from 1 and 5 s were the combination with more frequently highest accuracies. Classification using few features led to similar decision of the algorithms. Moreover, performance increased significantly according to the number of features used, reaching a plateau around 136. Finally, the results of this study suggested that *k*NN was the best algorithm to classify hand resting tremor in patients with PD.

## Introduction

More than 6.1 million people worldwide are affected by Parkinson’s disease (PD) (Gbd 2016 Parkinson’s Disease Collaborators, 2018) – this number is expected to rise with the increasing of the population life expectancy (Wanneveich et al., 2018). PD has very heterogeneous clinical features, but tremor at rest, akinesia, and rigidity are considered the clinical cardinal motor signatures of this disease (Kalia and Lang, 2015; Poewe et al., 2017). It is hard to diagnose PD, both in its early stages and during its progression. Its diagnosis is usually carried out by clinical observation or by using scales such as the Unified Parkinson’s Disease Rating Scale (UPDRS) or the Hoehn and Yahr scale (H-Y) (Hoehn and Yahr, 1967; Rizek et al., 2016; Holden et al., 2018).

Literature has proposed alternative ways to quantify PD symptoms in order to assist its diagnosis and progression (Jilbab et al., 2017). Inertial measures of the hand resting tremor associated to machine learning algorithms have been extensively investigated to distinct data from healthy people and patients with PD (Jeon et al., 2017a, b), to quantify the progression of the disease (Pedrosa et al., 2018), and to evaluate the effect of therapeutics on hands’ tremor (LeMoyne et al., 2019).

Although many investigations have evaluated the machine learning classifier performance to precisely categorize the inertial measurements from patients with PD, there are few methodological studies concerning the influence of the technical parameters of this kind of approach. Parameters like the time interval of the inertial sensor readings, type of features extracted from the inertial sensor readings, the number of features used, the type of machine learning classifier, and the type of inertial sensor used have potential to increase or decrease the accuracy of the algorithm (Jeon et al., 2017a; Rovini et al., 2017; Ramdhani et al., 2018; Wang et al., 2018; Nurwulan and Jiang, 2020). Table 1 lists examples of studies that associated inertial measurements with machine learning approaches and their methodological choices. It displays a large variability of methodological settings and few explanations justifying such choices.

**TABLE 1:** References that used inertial sensors features to feed machine learning to evaluate the hand tremor of PD patients.

References	Hand activity	Sensor (AR)	Recording duration	Methods of classification	Accuracy
Alam et al. (2016)	Resting tremor	Acc and gyros (200 Hz)	25–30 s	Support vector machine	59–88.9%
LeMoyne et al. (2015)	Kinetic tremor	Acc (100 Hz)	5 s	Support vector machine	100%
Butt et al. (2017)	Kinetic tremor	Gyros (100 Hz)	10 s	Support vector machine, logistic regression, neural network classifier	76.2–83.1%
Stamatakis et al. (2013)	Finger tapping	Acc (167 Hz)	Free	Ordinal logistic regression	87.2–96.5%
Jeon et al. (2017a)	Resting tremor	Acc (125 Hz)	10 s	SVM, decision tree, random forest, discriminant analysis	80.9–85.6

Several investigations have used a number of machine learning algorithms to classify and/or to quantify the resting hand tremor of patients with PD, obtaining high accuracy levels (Kostikis et al., 2015: 78–94%; Jeon et al., 2017a: 80–85%; Pedrosa et al., 2018: 92.8%). There is no consensus about what machine learning algorithms are preferable to classify features of inertial readings or what are the optimal conditions to use any of the algorithms.

Several studies have segmented inertial recordings in different window size durations to extract dozens or hundreds of features that fed a machine learning algorithm (Jeon et al., 2017a). Short-term inertial readings could be good to get a fast evaluation, but they lead to high false positive detection. On the other hand, long-term recordings may potentially prolong the recording process, adding redundant information (Nurwulan and Jiang, 2020). In the same way, using a few features may not be enough to bring clear information about the differences among patients with PD; and an excessive number of features may overload the computing process. It is important to select the best set of features in order to potentialize algorithm classification and to avoid collinearity among data.

The present study aimed to compare the performance of machine learning algorithms to classify recordings of inertial sensors as healthy people or patients with PD considering different numbers of features extracted from a variety of window length duration of inertial recordings. Those results may contribute in the decision making of the best parameter for the classification of inertial sensor measures analyzed by machine learning algorithms.

## Materials and Methods

### Ethical Considerations

All individual participants included in this study gave us their informed and written consent. Every procedure carried out in the present study was in accordance with the ethical standards of the Ethics Committee in Research with Humans from the University Hospital João de Barros Barreto (report #1.338.241) and with the 1964 Helsinki Declaration and its later amendments or comparable ethical standards.

### Subjects

Our sample comprised of 50 right-handed participants grouped into healthy control participants (*n* = 32 individuals, 16 females and 16 males) and participants with PD (*n* = 18 individuals, 8 females and 10 males). Participants’ handedness was established according to the hand they use to handwrite. Healthy participants ranged from 41 to 79 years (mean ± standard deviation: 64.3 ± 11.1 years), while patients with PD ranged from 48 to 73 years (mean ± standard deviation: 60.2 ± 8.4 years). Control participants were recruited by convenience. They had no history of neurological or systemic diseases, no self-reported tremor of the hands nor difficulties in carrying out daily activities. All patients with PD were diagnosed by a neurologist in the Neurology Department of the University Hospital João de Barros Barreto, Brazil, according to the clinical diagnostic criteria of the United Kingdom Parkinson’s Disease Society Brain Bank (Hughes et al., 1992). For each patient, the severity of PD was scored by using the Hoehn and Yahr (H-Y) scale. All patients with PD had disease diagnosed within the less 6 years; except by one subject (H-Y 3), all other patients were staged as functionally independent (H-Y 1 or 2). All patients were using levodopa or dopamine agonist therapy for over a year.

### Inertial Measurement Unit Recordings

**FIGURE 1:**
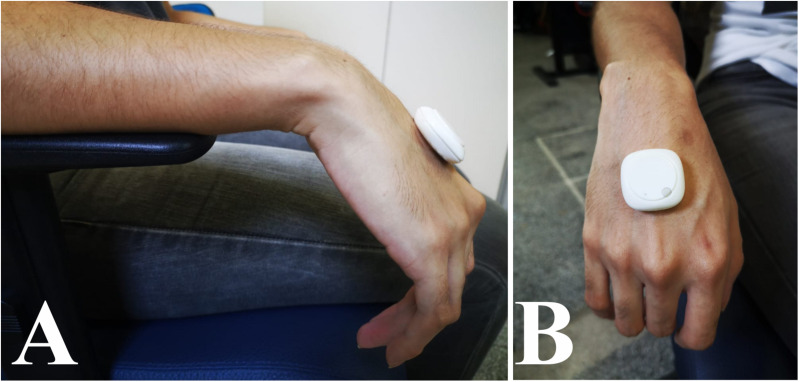
IMU Positioning in the hand of the participant. **(A)** Lateral view. **(B)** Frontal view. The patient was instructed to keep the hand in rest for 120 s, while the experimenter controlled the recording using a mobile app.

We used a wearable device MetaMotionC (mbientlab, San Francisco, United States), with on-board sensors, such as a triple-axis gyroscope and an accelerometer (16 bits, ± 2000°/s, ± 16 g). Researchers positioned a wearable device over each patient’s third metacarpal bone at their midway between the carpal and the digital extremities of their metacarpal (Figure 1) – with their forearm supported on a table, and their hand relaxed over its edge. Researchers recorded the patients in resting state with the acquisition rate at 100 Hz and 16-bit analog to digital conversion resolution. An Android app (MetaBase, mbientlab, United States) controlled the sensors via Bluetooth. Bluetooth also transmitted their signals to an ordinary computer. The study delivered 2-min recordings. One trial was carried out for each one of the hands of all participants.

### Data Analysis

To carry out data analysis, researchers programmed Python scripts (Python v3.7.4) by using SciPy (version 1.3.1), NumPy (version 1.17.2), PyWavelets (version 1.0.3), and LibROSA (version 0.7.2) tools. SciPy is a Python-based ecosystem of open-source software for mathematics, science, and engineering; NumPy is a library for the Python programming used to operate on arrays; LibROSA is a Python package that provides the building blocks necessary to create music information retrieval systems; and PyWavelets is an open source wavelet that transforms software for Python.

**FIGURE 2:**
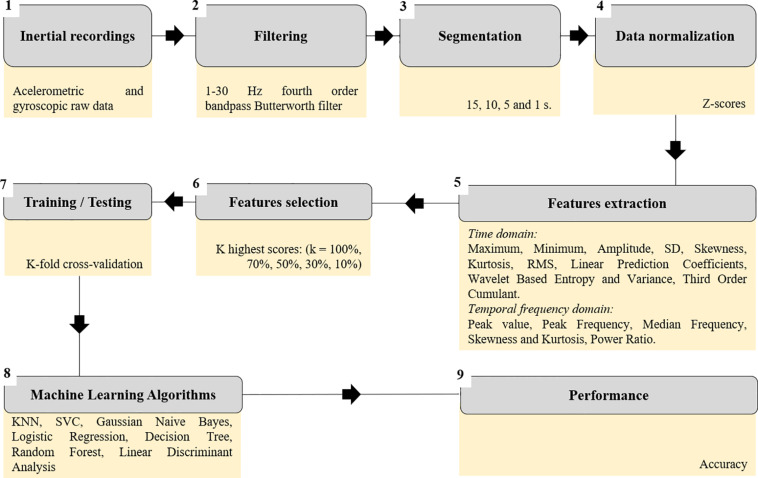
Flow chart of the data analysis steps.

Our sequence of analysis consisted of: (1) inertial recordings; (2) raw data filtering; (3) segmentation of the time series in different sets of waveform lengths; (4) data normalization; (5) extraction of features; (6) selection of the best features; (7–8) performing machine learning algorithms with training and test phases; and (9) measuring machine learning performance. Figure 2 illustrates data analysis summary.

#### Raw Data Filtering

We computed a magnitude vector from each sensor dimension (x, y, and z) using Eq. (1), which is less sensitive to orientation changes (Janidarmian et al., 2017). The recordings were filtered by a fourth-order bandpass digital Butterworth filter between 1 and 30 Hz to exclude low and high frequency artifacts.

(1) $$\begin{array}{c} v=\sqrt{x^{2}+y^{2}+z^{2}} \end{array}$$

where *v*is the magnitude vector, *x*,*y*, and *z* represented the 3-D readings of the inertial sensor.

After this, we applied the *scipy.signal.detrend* function using its linear list squared fit to detrend the inertial readings.

#### Segmentation of the Time Series

We segmented the inertial recordings in fixed sized windows, with no inter-window gaps and non-overlapping between adjacent windows. We also segmented these time series in sets of waveforms with 1, 5, 10, and 15 s window sizes.

#### Extraction of Features

We extracted features from time and temporal domains for each sensor dimension. Table 2 presents a list of features extracted from inertial data, as well as Python main codes related to them.

**TABLE 2:** Features extracted from the inertial readings.

Features	Python code
**Time domain**	
Range	range = values.max() - values.min()
Standard deviation	std = values.std()
Root mean square	rms = numpy.sqrt(numpy.mean(values^∗∗^2))
Skewness	sk = scipy.stats.skew(values)
Kurtosis	kt = scipy.stats.kurtosis(values)
Linear prediction coefficients	lp_coefs = librosa.lpc(values, 3)
Wavelet transform detail coefficients (cD)	_, cD = pywt.dwt(values, ’db3’)
cD variance	variance = numpy.var(cD)
cD entropy	def approximate_entropy(U, m = 2, r = 3):
	U = numpy.array(U)
	N = U.shape[0]
	def phi(m):
	z = N - m + 1.0
	x = numpy.array([U[i:i + m] \
	for i in range(int(z))])
	x_ = numpy.repeat(x[:, \
	numpy.newaxis], 1, axis = 2)
	C = numpy.sum(numpy.absolute(x - \
	x_).max(axis = 2) < = r, \
	axis = 0)/z
	return numpy.log(C).sum()/z
	entropy = abs(phi(m + 1) - phi(m))
Third order cumulant	third_order_cum = scipy.stats.moment(values, moment = 3)
**Temporal frequency (tf) domain**	
Peak of energy	p_tf = frequency_values.max()
Frequency at the peak energy	xf = numpy.linspace(0, af/2,
	frequency_values.size)
	tf_p = xf[numpy.argmax(frequency_values)]
Skewness_tf	sk_tf = scipy.stats.skew(frequency_values)
Kurtosis_tf	kt_tf = scipy.stats.kurtosis(frequency_values)
Mean frequency	def mean_frequency(frequency_values):
	xf = numpy.linspace(0, af/2,
	frequency_values.size)
	xf = xf[xf > = 1]
	total_area = numpy.trapz(frequency_values, xf)
	for i, x in enumerate(xf):
	partial_area = numpy.trapz(frequency_values[:i],
	xf[:i])
	if partial_area > total_area/2:
	mean_freq = xf[i-1]
Power ratio (1–6 Hz/6–12 Hz)	xf = numpy.linspace(0, af/2,
	frequency_values.size)
	num = frequency_values[(xf > = 1) &
	(xf < = 6)]
	den = frequency_values[(xf > = 6) &
	(xf < = 12)]
	power_ratio = num.mean()/den.mean()

*values, inertial measures in the time domain vector; frequency_values, inertial measures in the temporal frequency domain vector; af, the acquisition frequency; and, xf, frequency values vector.*

The study extracted 272 features from each one of our participants, considering data extracted: (a) from each one of their hands (dominant and non-dominant); (b) from each inertial sensor parameter (accelerometer and gyroscope); and, (c) from the four dimensions of each sensor (*x*, *y*, *z*, and magnitude).

#### Data Normalization

The study applied *sklearn.preprocessing* package and its *StandardScaler* function to standardize features by removing their mean and scaling them to unit variance, as shown in Eq. (2).

(2) $$\begin{array}{c} z\,\_\,s\,c\,o\,r\,e=\frac{\left(x-\mu \right)}{s} \end{array}$$

#### Selection of Features

The study used algorithm *SelectKBest* to select the k most important features based in a score which was the ANOVA *F*-value. The chosen selection of the most important features to feed the machine learning algorithms in this study where: 272 features (100%), 190 features (70%), 136 features (50%), 82 features (30%), and 27 features (10%).

#### Splitting Data

To validate the predictive models, we applied the tenfold cross-validation method by using the *Scikit-learn* library (version 0.21.3) and *ShuffleSplit* function. The study randomly split data into 80% for model training and 20% for model testing.

#### Machine Learning Algorithms

We applied seven types of machine learning algorithms to classify the data from both healthy and PD groups. The algorithms were: *k*-nearest-neighbor (*k*NN); support vector classifier (SVC); logistic regression (LR); linear discriminant analysis (LDA); random forest (RF); decision tree (DT); and Gaussian Naïve Bayes (GNB).

The next sentences describe the Python functions used to proceed the machine learning algorithms, as well as the parameters that differed from default values. These parameters were changed to protect the model from overfitting.

(a) *k*-Nearest-Neighbor (*k*NN): the function *sklearn.neighbors.KNeighborsClassifier* was applied to proceed an *k*NN algorithm considering the Minkowski distance metrics, *k*-value ranging from 5 to 10. We applied a grid search using the *GridSearchCV* function to find which *k*-nearest-neighbor would deliver the best accuracy, then chosen as the best *k*-value.
(b) Support Vector Classifier (SVC): were applied an SVC algorithm (*sklearn.svm.SVC* function) with radial basis function kernel with *gamma* parameter equal to 1 and the *C penalty* parameter equal to 10.
(c) Logistic Regression (LR): a binary logistic regression algorithm *sklearn.linear_model.LogisticRegression* function was used considering the parameter *penalty* equal to “l1,” and *solver* equal to “liblinear.”
(d) Linear Discriminant Analysis (LDA): the study applied the function *sklearn.discriminant_analysis.LinearDiscriminantAnalysis* to proceed the LDA algorithm considering the parameter *solver* equal to “svd,” and *store_covariance* as true.
(e) Random Forest (RF): we used the function *sklearn.ensemble.RandomForestClassifier* to implement random forest algorithm considering the parameter “*criterion”* the value “*gini impurity*” as a measure of the split quality, the parameters *n_estimators* equal to 50, and *max_depth* equal to 6.
(f) Decision Tree (DT): similarly to the random forest classifiers, the tree algorithm was proceed using the *sklearn.tree.DecisionTreeClassifier* function considering “*gini impurity*” to the parameter “*criterion*,” and the parameters *n_estimators* were set to 50, and *max_depth* equal to 6.
(g) Gaussian Naïve Bayes (GNB): the function to proceed a Gaussian Naïve Bayes algorithm was the *sklearn.naive_bayes.GaussianNB*.

#### Measuring Machine Learning Performances

Equation (3) calculated accuracy in order to measure the success levels of the classifiers, as follows:

(3) $$\begin{array}{c} A\,c\,c\,u\,r\,a\,c\,y=\frac{\left(T\,P+T\,N\right)}{\left(T\,P+F\,P+T\,N+F\,N\right)} \end{array}$$

where TP is the true positive value; TN is the true negative value; FP is the false positive value; and, FN is the false negative value.

### Statistics

The study applied the unpaired *t*-test with Welch’s correction to compare the accuracies obtained from training and testing phases for each classifier using features extracted from different time window lengths. For each percentage of features feeding the algorithms, we conducted a two-way ANOVA on the influence of the classifier type and the time window length of the accuracy of such classifier. The classifier type includes seven levels (SVC, GNB, RF, *k*NN, LR, LDA, and DT) and the time window length consisted of five levels (1, 5, 10, and 15 s). As the two-way ANOVA test was significant, we computed the Tukey HSD for performing multiple pairwise-comparison between mean accuracies of both groups. We counted the number of times in which an algorithm presented a better performance when compared to the others (here named victory), by means of significant multiple comparisons at the different time window lengths and number of features. Thus, we used the chi-square goodness of fit (equal proportions) to compare the observed distribution of significant comparisons to the expected distribution considering the number of algorithms or of time window length. All the statistical tests were carried out by using R software (version 3.6) and considering the level of significance of 5%.

## Results

### Selection of Recordings and Features

**FIGURE 3:**
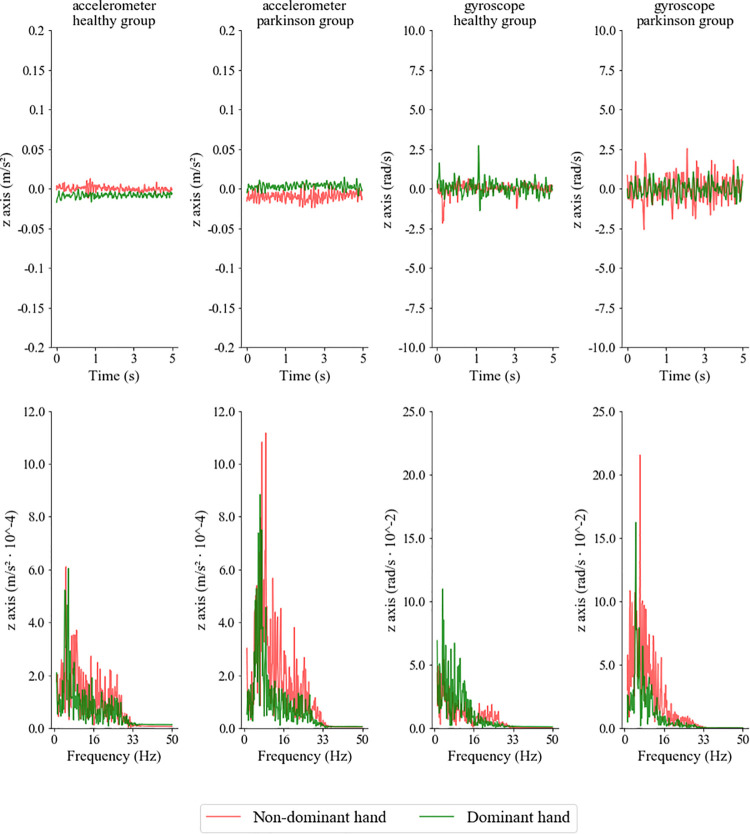
Accelerometric and gyroscopic recordings as a function of the time (upper rows) and temporal frequency (lower row) from representative participants of the control and PD groups, using the time window of 5 s. Recordings were carried out on the non-dominant and dominant hands (red and green lines, respectively).

Figure 3 shows examples of the accelerometric and gyroscopic recordings for the 5-s time windows as a function of time and temporal frequency from representative subjects from both groups. The results for the 5 s time windows were qualitatively similar to the other time windows the study investigated. We characterized the inertial recordings by oscillatory waveforms that, especially in participants with PD, defined their peak in frequencies ranging between 3 and 8 Hz.

**FIGURE 4:**
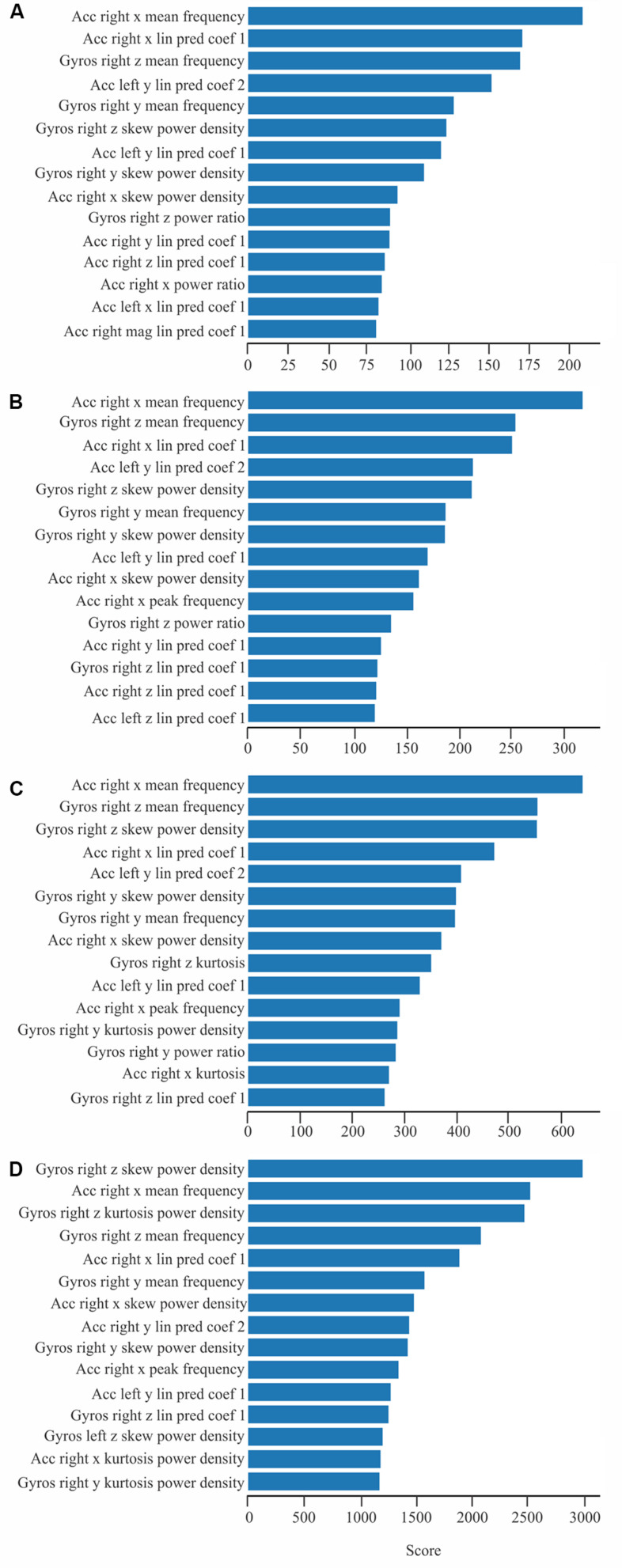
Most important features extracted from recordings lasting 1 s **(A)**, 5 s **(B)**, 10 s **(C)**, and 15 s **(D)**.

Regardless time window length, the most important features detected were mean frequency, linear prediction coefficients, power ratio, and the power density skew and kurtosis. Figure 4 shows the 15 most important features selected from extracted data concerning time windows of 15 s (Figure 4A), 10 s (Figure 4B), 5 s (Figure 4C), and 1 s (Figure 4D).

### Machine Learning Classifiers

#### Comparison Between Training and Testing Accuracies

Most of the comparisons had significant differences between training and testing phases. Whenever statistical significance (*p* < 0.05) was reached, testing accuracy was higher than training accuracy – except in two comparisons (random forest and *k*NN algorithms) – when using 30% of the features in the 1 s time window. Supplementary Files 1–5 present tables with the training and testing phases of the machine learning.

The comparisons with no statistical significance were in time windows of:

(i) 1 s: random forest algorithm using all features and 70% of them, GNB using 50 and 10%;
(ii) 5 s: GNB with all features, 70 and 50% of them, *k*NN and LR using 30% of the features;
(iii) 10 s: GNB using 30 and 10% of the features;
(iv) 15 s: GNB using all features, 70, 50, and 10% of them, SVC using all features, 70 and 50% of them, LDA using all features and 70% of them, LR using 50% of the features, and RF using 30% of the features.

**FIGURE 5:**
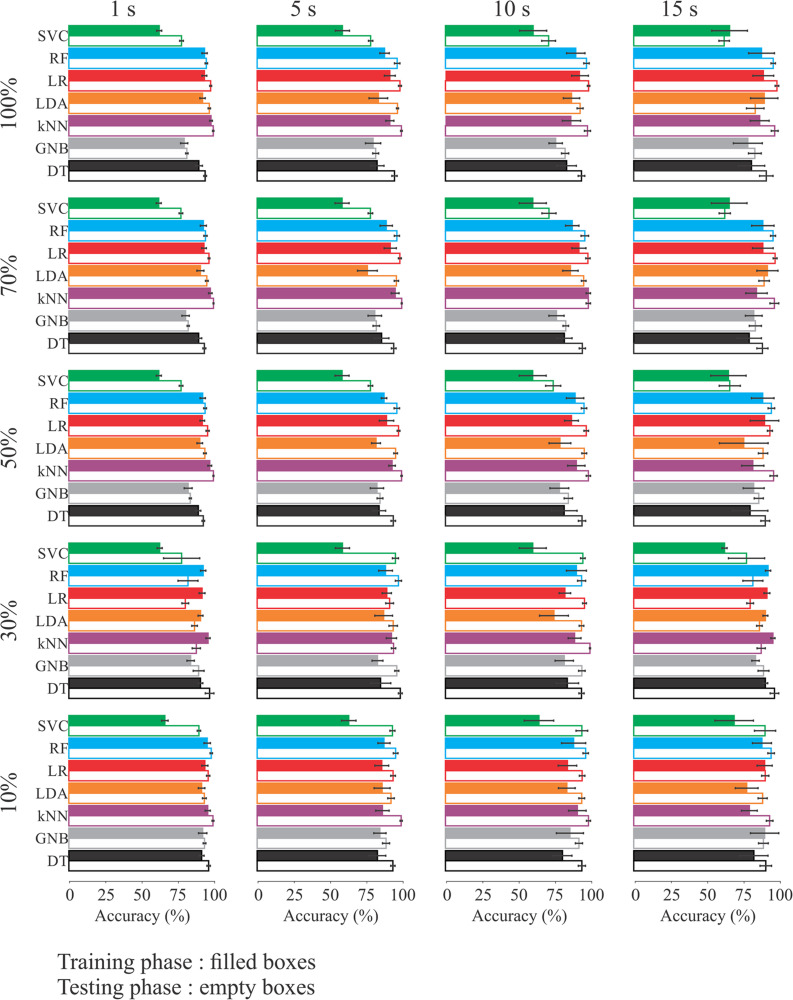
Comparison of the classifiers’ performance in the training (solid bars) and testing (empty bars) phase according the number of features and time window length.

Figure 5 illustrates the comparisons between the accuracies obtained by the different classifiers using extracted features in different time windows considering 70, 50, 30, and 10% of the features, respectively.

#### Comparing Test Accuracies Obtained From the Different Supervised Machine Learning Algorithms

In general, the effects of the machine learning phases on the accuracies were statistically significant. The main effect for classifier type yielded an *F* ratio of *F*(6, 252) = 639.14, *p* < 0.0001 for all the features; *F*(6, 252) = 727.74, *p* < 0.0001 for 70% of the features; *F*(6, 252) = 478.15, *p* < 0.0001 for 50% of the features; *F*(6, 252) = 171.41, *p* < 0.0001 for 30% of the features; and *F*(6, 252) = 36.8, *p* < 0.0001 for 10% of the features. The proportion of victories in the multiple comparisons significantly differed by algorithm for all numbers of features conditions. *k*NN was the algorithm that more frequently delivered high accuracy when compared to the others algorithms. SVC delivered the lowest frequency of victories among all tested algorithms. Table 3 shows the number of “victories” of each algorithm in the significant multiple comparisons for each number of feature condition.

**TABLE 3:** Number of victories of each classifier in the significant multiple comparisons for each number of feature condition.

	Number of features				
Algorithm	100%	70%	50%	30%	10%
SVC	5	5	3	0	4
GNB	12	16	16	13	2
RF	40	40	39	31	27
*k*NN	54	58	61	50	50
LR	53	48	41	31	6
LDA	34	38	35	27	3
DT	36	37	34	28	5
Number of significant multiple comparisons	234	242	229	180	97
*X*^2^	63.53	57.72	63.50	57.38	142.51
*P*	<0.0001	<0.0001	<0.0001	<0.0001	<0.0001

The main effect for time window length yielded an *F* ratio of *F*(3, 252) = 51.7, *p* < 0.0001 for all the features; *F*(3, 252) = 47.4, *p* < 0.0001 for 70% of the features; *F*(3, 252) = 25.5, *p* < 0.0001 for 50% of the features; *F*(3, 252) = 5.5, *p* < 0.0001 for 30% of the features; and *F*(3, 252) = 14.8, *p* < 0.0001 for 10% of the features. The proportion of victories in the multiple comparisons was similar by time window length for all numbers of feature conditions, except for 10% of the features. Table 4 displays the number of “victories” from time window length in the significant multiple comparisons for each number of feature condition.

**TABLE 4:** Number of victories per time window length in the significant multiple comparisons for each number of feature condition.

	Number of features				
Time window length	100%	70%	50%	30%	10%
1 s	58	61	54	39	12
5 s	64	68	66	52	35
10 s	60	62	60	47	27
15 s	52	51	49	42	23
Number of significant multiple comparisons	234	242	229	180	97
*X*^2^	1.28	2.46	2.84	2.17	11.33
*P*	0.73	0.48	0.51	0.53	<0.01

The interaction effect was significant for all numbers of features conditions (for all the features: *F*(18, 252) = 19.04, *p* < 0.001; for 70% of the features: *F*(18, 252) = 15.23, *p* < 0.001; For 50% of the features: *F*(18, 252) = 7.61, *p* < 0.001; and for 10% of the features: *F*(18, 252) = 2.959, *p* < 0.001), except for 30% of the features condition that yielded in a *F* ratio of *F*(18, 252) = 2.959, and *p* = 0.29.

**FIGURE 6:**
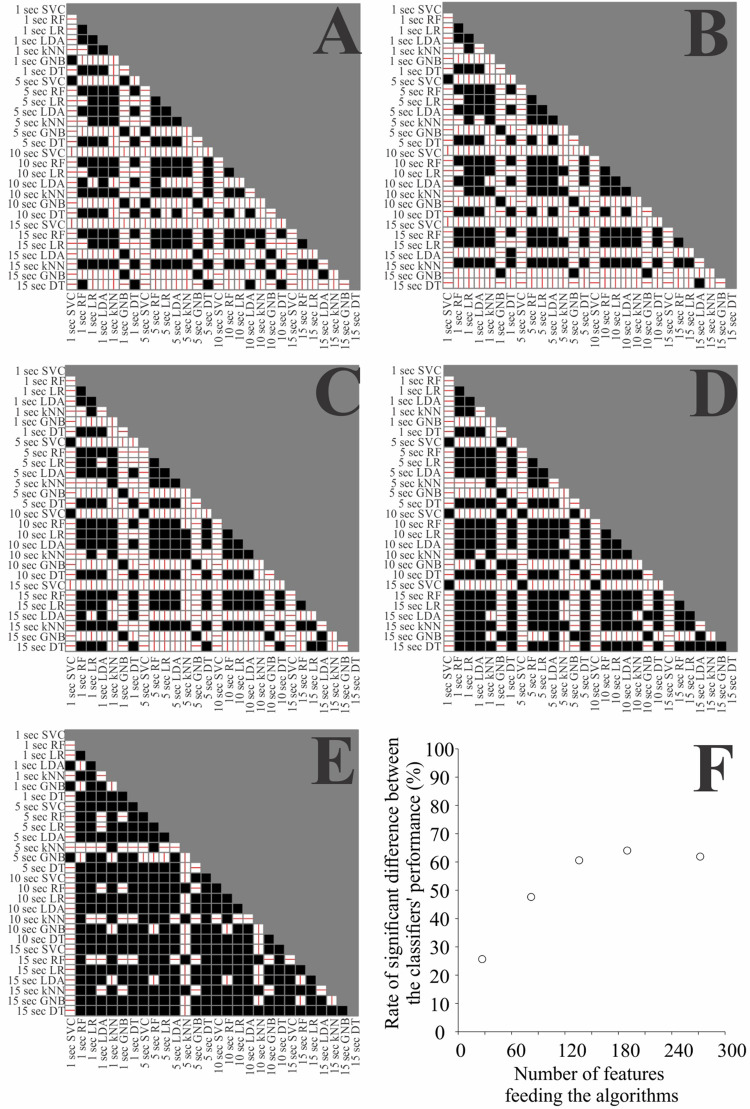
Comparison of the classifier’s performance in the testing phase when using all the features **(A)**, 70% **(B)**, 50% **(C)**, 30% **(D)**, and 10% **(E)** of the features. White squares represent the significant difference between the classifiers on the respective row and column, while black squares represent non-significance for the comparison. The line in the white squares represent the direction of the difference, horizontal lines indicates that the classifier on the row had higher accuracy than the classifier on the column, and vertical lines represent the opposite. **(F)** Number of significant differences between two classifiers as a function of number of features.

Figures 6A–E shows tile plots representing the statistical significance of the *post hoc* multiple comparisons between the testing accuracies from any two classifiers. White tiles represent comparisons with significant differences, while dark tiles represent non-significant differences. The red line indicates the orientation of the significant difference. Horizontal lines represent higher accuracies for the classifiers in the row when compared to the classifiers in the column, while vertical lines represent the opposite situation. We observed that the number of significant differences between two classifiers (number of white tiles) was dependent of the number of features. For a low number of features (10% of the features we extracted, 27 features) the number of significant differences between two classifiers was also low and increased linearly up to reach a plateau level of 70% of the features (136 features). The combinations between classifier and time window length with highest accuracies were *k*NN and time windows of 1 and 5s.

## Discussion

This paper assessed the hand tremor in individuals with PD and healthy controls by using machine learning algorithms based on inertial sensor recordings. Our objectives were: (i) identifying the best machine learning algorithms to classify hand tremor by using inertial data; (ii) describing the best recording duration to be used by classification methods; (iii) stablishing the number of features necessary to the best performance of the algorithms.

Concerning these objectives, the results of this study showed that the *k*NN algorithm as the best classifier, followed by LR, and RF algorithms respectively. On the other hand, research pointed out that SVC and GNB delivered the worst performances among all classifiers. Also, some classifiers had better performances with short time windows, while others needed long recordings to deliver more accurate performances. Our results also showed that the performance of the classifiers became more similar when using less features; and, with more features, differences between classifiers increased linearly until a maximum value (using around 136 features), reaching a plateau. Regardless the most important feature selected, the time window length was similar across tested conditions. Whereas, the more common features selected were mean frequency for both accelerometer and gyroscope sensors; linear prediction coefficients for the accelerometer; skewness, power ratio, and the power density skewness and kurtosis for the gyroscope.

Many types of machine learning classifiers have been used to analyze PD tremor (Bind et al., 2015). We used 7 out of the most common algorithms used in the field. *k*NN was the best classifier across multiple comparisons, together with LR and RF algorithms, which had accuracy level above 90%.

The *k*NN algorithm groups similar classes of data based in the value of *k* nearest neighbors. Low values of *k* increase the accuracy of the classifier in the training phase, but difficult the generalization of the model for a new data (Li and Zhang, 2011). The *k* was used between 5 and 10 to facilitate the generalization of the model during test phase. Previous investigations – such as Jeon et al. (2017b) – have also found high accuracies using *k*NN algorithms. They assessed 85 PD patients to predict UPDRS results by using a wrist-watch-type wearable device for measuring tremors and found an accuracy level close to 84% for *k*NN and RF algorithms. Also, *k*NN algorithm delivered performance improvement as we decreased the number of features, while other algorithms delivered impaired outcomes.

Random Forest is a combination of multiple tree predictors that make decisions based in random vectors of features. The RF decision is the more common decision of the collection of tree classifiers (Breiman, 2001). Previous studies have demonstrated the ability of RF models to detect freezing in the gait of patients with PD or the switching on and off state of deep brain stimulation in these patients (Tripoliti et al., 2013; Kuhner et al., 2017).

Logistic Regression is a classification algorithm that uses a logistic sigmoid function to transform observations in two or more classes. LeMoyne et al. (2019) used LR algorithms to distinguish inertial readings associated with on and off modes from deep brain stimulation in PD patients, getting an accuracy level of 95%.

Both GNB and SVC with the worst outcomes. When compared with other algorithms, the GNB classifier delivered lower (Susi et al., 2011) and higher (Bazgir et al., 2018) accuracies to detect human motion. GNB is an algorithm that evaluates the probability of events within different classes (Theodoridis et al., 2010; Bazgir et al., 2018). SVC aims to find an optimal separation hyperplane in order to minimize misclassifications (Vapnik, 1979). SVC has been widely used to detect tremor in PD patients. The accuracy level of its classifiers has ranged between 80 and 90% to quantify PD tremor (Alam et al., 2016; Jeon et al., 2017b). We used a radial compared to the best SVC used by Jeon et al. (2017b) finding similar results.

It is important to highlight that directly comparing the performance of the classifiers in different studies must be careful. Each study implements different parameters in the algorithms, which are not always fully described. Furthermore, the number and type of features may influence the classifier accuracies. The present study observed that few features make classifiers’ decisions more similar, while an increased number of features enable the classifiers’ performance to be distinguished, reaching a plateau around 176 features. One must find a trade-off between the number of features and the cost of computational processing for each algorithm especially when trying to implement such method with wearable or mobile devices.

The use of machine learning algorithms to recognize patterns of human motion requires the segmentation of motion recording time series. Previous studies have segmented time series in different lengths for pattern recognition tasks (Bussmann et al., 2001; Dehghani et al., 2019). Although, short lengths accelerate the duration of the recordings, their random nature can present negative influence on the classifiers’ performance (Mannini et al., 2013). Short duration recordings in the scale of 100 ms have been successfully used to recognize human motion. At the same time, long-term recordings also returned high accuracy when detecting PD tremor as we can observe in Table 1.

This study evaluated the accuracy of classifiers by using different time window lengths. We observed that recordings lasting 5s or 1s delivered the highest accuracy levels. The study also noticed some interaction between the window time length and classifiers, indicating that some classifiers were better to analyze short recordings (i.e., *k*NN algorithm), while others showed higher accuracies when using long recordings (i.e., GNB). There is no rule concerning the length of inertial readings for the predictive modeling problem. Banos et al. (2014) investigated the effects of the windowing procedures on the activity recognition process using inertial data. They observed that intervals between 1 and 2 s offered the best trade-off between recognition speed and accuracy.

The more common features extracted from inertial readings express amplitude of oscillatory series, their spectral content, regularity, and coherence (Meigal et al., 2012; Twomey et al., 2018). The present study observed that mean frequency for both accelerometer and gyroscope sensors, linear prediction coefficients for the accelerometer, and skew power ratio, and the power density skew and kurtosis for the gyroscope frequently figure among the fifteen top features. Frequency domain features have been successfully employed in the machine learning algorithms by other researchers (Bazgir et al., 2018; Pedrosa et al., 2018).

We based our approach exclusively on accelerometer and gyroscope sensors, though other sensors are reported in the literature to quantify PD hand tremor using machine learning algorithms. For example, Lonini et al. (2018) used the MC10 BioStampRC sensor, a sensor tape that records electromyographic signals to accelerometers and gyroscopes in 6 body positions. Even considering that additional sensors can contribute to increase the accuracy of a classifier, there is a high cost in its implementation that can reduce the applicability of the proposal. Inertial sensors are inexpensive instruments that are available in a wide variety of wearable equipment.

This study has some potential limitations that deserve further comments. To date, research on this topic has been exploratory. There are no guidelines regarding the use of machine learning approach to quantify hand tremor in PD patients, as well as no established parameters for the choice of inertial sensors. A larger sample size and longitudinal follow-up could reinforce the present interpretations.

## Conclusion

The present study suggested *k*NN using hundreds of features extracted from short-term inertial recordings as the best settings for machine learning configuration to classify hand tremor in PD patients. Our results can be used to assist the diagnosis and follow up of PD patients. We consider that our results are robust, because (i) of the high accuracy level obtained with the classifiers, (ii) the study could separate patients in the early stage of the PD (low H-Y score) from healthy people.

## Data Availability Statement

All datasets generated for this study are included in the article/Supplementary Material.

## Ethics Statement

The studies involving human participants were reviewed and approved by the Ethics Committee in Research with Humans from the University Hospital João de Barros Barreto. The patients/participants provided their written informed consent to participate in this study.

## Author Contributions

GS, AK, GP, and AAC conceived of the presented idea. ES and GP performed the computations. AA, KS, VF, FS, and RL collected the inertial recordings. LK and BS-L collected the clinical data. AA, ES, GS, AAC, and BC verified the analytical methods. ASC and AB contributed to the interpretation of the results. GS and AAC drafted the manuscript. All authors discussed the results and contributed to the final manuscript.

## Conflict of Interest

The authors declare that the research was conducted in the absence of any commercial or financial relationships that could be construed as a potential conflict of interest.
