# Supplementary material for: Hand Resting Tremor Assessment of Healthy and Patients With Parkinson’s Disease: An Exploratory Machine Learning Study
Source: Front Bioeng Biotechnol. 2020 Jul 14;8:778. doi: 10.3389/fbioe.2020.00778 (PMC7381229; doi:10.3389/fbioe.2020.00778)
Supplement: Supplementary file 1 [file Table_1.DOCX]

| **Classifiers** | **Training phase** | **Testing phase** | **p-value** |
| --- | --- | --- | --- |
| *Window length of 1 s* |  |  |  |
| SVC | 61.2±1.7 | 76.8±1.2 | 0.0001 |
| Gaussian NB | 78.6±2.4 | 80.5±0.7 | 0.0386 |
| RF | 92.5±1.8 | 93.7±0.8 | 0.0668 |
| *k*NN | 96.9±0.9 | 98.4±0.4 | 0.0002 |
| LR | 92.4±1.6 | 96.7±0.6 | 0.0001 |
| LDA | 91.2±1.8 | 95.8±0.7 | 0.001 |
| DT | 88.6±2.4 | 93±0.9 | 0.001 |
| *Window length of 5 s* |  |  |  |
| SVC | 58.5±4.8 | 77.9±1.5 | 0.0001 |
| Gaussian NB | 79.6±5.2 | 81.4±2 | 0.3252 |
| RF | 87.4±3.2 | 96.1±1.8 | 0.0001 |
| *k*NN | 91.1±2.8 | 99 ±0.5 | 0.0001 |
| LR | 91.1±3.7 | 98 ±1 | 0.0001 |
| LDA | 83.2±6.4 | 96.3±0.6 | 0.0001 |
| DT | 82.3±4.7 | 94.3±1.8 | 0.0001 |
| *Window length of 10 s* |  |  |  |
| SVC | 60±9.3 | 70.6±4.7 | 0.0047 |
| Gaussian NB | 75.4±4.6 | 81.9±2.4 | 0.001 |
| RF | 89.2±6.3 | 96.5±1.9 | 0.0023 |
| *k*NN | 86.3±6.2 | 97.2±2.1 | 0.0001 |
| LR | 92.1±5.7 | 97.9±0.8 | 0.0048 |
| LDA | 86.3±0.06 | 92.1±2 | 0.0057 |
| DT | 82.9±6.6 | 93.1±2.3 | 0.0002 |
| *Window length of 15 s* |  |  |  |
| SVC | 65.6±9.8 | 61.9±3.6 | 0.3706 |
| Gaussian NB | 78.1±4.8 | 82.9±4.3 | 0.1749 |
| RF | 87.5±8.8 | 95.5±1.5 | 0.0112 |
| *k*NN | 86.3±6.5 | 96.5±2.3 | 0.0002 |
| LR | 88.8±7.1 | 98.1±1.2 | 0.0007 |
| LDA | 89.4±9.3 | 83.2±6 | 0.0958 |
| DT | 80.6±9.1 | 90.8±4.6 | 0.0054 |

**Supplementary Table 1.** Comparison of the accuracies (mean ± standard deviation) calculated from training and testing phases considering the different time window lengths using all the extracted features.
