# Supplementary material for: Hand Resting Tremor Assessment of Healthy and Patients With Parkinson’s Disease: An Exploratory Machine Learning Study
Source: Front Bioeng Biotechnol. 2020 Jul 14;8:778. doi: 10.3389/fbioe.2020.00778 (PMC7381229; doi:10.3389/fbioe.2020.00778)
Supplement: Supplementary file 2 [file Table_2.DOCX]

| **Classifiers** | **Training phase** | **Testing phase** | **p-value** |
| --- | --- | --- | --- |
| *Window length of 1 s* |  |  |  |
| SVC | 61.6±1.7 | 76.8±1.2 | 0.0001 |
| Gaussian NB | 80±2.5 | 81.8±0.7 | 0.0441 |
| RF | 92.2±2.1 | 93.7±1.1 | 0.0658 |
| *k*NN | 97±1.2 | 99.2±0.3 | 0.0001 |
| LR | 92.6±1.6 | 96.1±0.6 | 0.0001 |
| LDA | 90.2±2.4 | 94.6±0.9 | 0.0001 |
| DT | 89±2.1 | 92.9±1 | 0.0001 |
| *Window length of 5 s* |  |  |  |
| SVC | 58.5±4.8 | 78±1.6 | 0.0001 |
| Gaussian NB | 81.1±4.6 | 82.1±2.2 | 0.5355 |
| RF | 88.9±4.2 | 96.3±1.8 | 0.0001 |
| *k*NN | 95.1±2.7 | 99.6±0.3 | 0.0001 |
| LR | 91.7±4.2 | 98.3±1 | 0.0001 |
| LDA | 76±6.8 | 95.9±1.5 | 0.0001 |
| DT | 85.7±5 | 94.1±1.6 | 0.0001 |
| *Window length of 10 s* |  |  |  |
| SVC | 60±9.3 | 71.1±4.8 | 0.0034 |
| Gaussian NB | 76.3±5.2 | 82.6±1.9 | 0.0018 |
| RF | 87.1±4.6 | 95.7±2.6 | 0.0001 |
| *k*NN | 88.3±5.8 | 98.1±1.5 | 0.0001 |
| LR | 91.7±4.8 | 97.9±1.5 | 0.0011 |
| LDA | 85.8±5.3 | 95±1.6 | 0.0001 |
| DT | 81.7±5.3 | 94±2.1 | 0.0001 |
| *Window length of 15 s* |  |  |  |
| SVC | 65.6±12.2 | 62.6±3.9 | 0.4604 |
| Gaussian NB | 82.5±5.7 | 83.6±4.1 | 0.6322 |
| RF | 88.8±7.7 | 95.8±1.7 | 0.0113 |
| *k*NN | 84.4±7.4 | 96.7±3 | 0.0001 |
| LR | 88.8±7.1 | 97.2±1.3 | 0.0017 |
| LDA | 91.9±7.2 | 89.6±3.6 | 0.3888 |
| DT | 79.4±8.4 | 88.5±3.8 | 0.0058 |

**Supplementary Table 2.** Comparison of the accuracies (mean ± standard deviation) calculated from training and testing phases considering the different time window lengths using 70% of the extracted features.
