# Supplementary material for: Hand Resting Tremor Assessment of Healthy and Patients With Parkinson’s Disease: An Exploratory Machine Learning Study
Source: Front Bioeng Biotechnol. 2020 Jul 14;8:778. doi: 10.3389/fbioe.2020.00778 (PMC7381229; doi:10.3389/fbioe.2020.00778)
Supplement: Supplementary file 3 [file Table_3.DOCX]

| **Classifiers** | **Training phase** | **Testing phase** | **p-value** |
| --- | --- | --- | --- |
| *Window length of 1 s* |  |  |  |
| SVC | 61.2±1.7 | 76.8±1.2 | 0.0001 |
| Gaussian NB | 81.7±2.7 | 83.1±0.7 | 0.1157 |
| RF | 91.6±1.3 | 93.3±0.9 | 0.0196 |
| *k*NN | 96.4±1.3 | 99±3.2 | 0.0001 |
| LR | 91.4±1.6 | 95±1.1 | 0.0001 |
| LDA | 89.7±1.9 | 93.2±0.9 | 0.0001 |
| DT | 88.7±1.7 | 92.1±0.8 | 0.0001 |
| *Window length of 5 s* |  |  |  |
| SVC | 58.5±4.8 | 78.1±1.5 | 0.0001 |
| Gaussian NB | 82.6±0.46 | 84.7±2 | 0.199 |
| RF | 87.4±1.9 | 96.3±1.9 | 0.0001 |
| *k*NN | 93±2.3 | 99.5±0.4 | 0.0001 |
| LR | 81.4±5 | 97.4±0.9 | 0.0001 |
| LDA | 81.9±3.1 | 95.4±1.2 | 0.0001 |
| DT | 84±4.5 | 93.8±1.5 | 0.0001 |
| *Window length of 10 s* |  |  |  |
| SVC | 60±9.3 | 74.1±5.2 | 0.005 |
| Gaussian NB | 78.3±6.5 | 84.5±2.9 | 0.0126 |
| RF | 89.2±0.6 | 95.3±1.9 | 0.0061 |
| *k*NN | 90±6 | 98.3±1.4 | 0.0005 |
| LR | 86.7±4.7 | 96.8±1.6 | 0.0001 |
| LDA | 78.8±7.5 | 95.5±1.7 | 0.0001 |
| DT | 81.7±8.8 | 93.9±2.5 | 0.0005 |
| *Window length of 15 s* |  |  |  |
| SVC | 65.6±12.2 | 66.5±7.3 | 0.8416 |
| Gaussian NB | 83.1±7.2 | 86.7±3.1 | 0.1735 |
| RF | 89.3±7.8 | 95.4±2.3 | 0.0315 |
| *k*NN | 82.5±7.7 | 96.9±2.5 | 0.0001 |
| LR | 90.6±9.9 | 94.4±1.7 | 0.2545 |
| LDA | 76.3±0.2 | 89.6±3.2 | 0.0242 |
| DT | 80.6±0.1 | 91.2±3 | 0.0171 |

**Supplementary Table 3.** Comparison of the accuracies (mean ± standard deviation) calculated from training and testing phases considering the different time window lengths using 50% of the extracted features.
