# Supplementary material for: Hand Resting Tremor Assessment of Healthy and Patients With Parkinson’s Disease: An Exploratory Machine Learning Study
Source: Front Bioeng Biotechnol. 2020 Jul 14;8:778. doi: 10.3389/fbioe.2020.00778 (PMC7381229; doi:10.3389/fbioe.2020.00778)
Supplement: Supplementary file 4 [file Table_4.DOCX]

| **Classifiers** | **Training phase** | **Testing phase** | **p-value** |
| --- | --- | --- | --- |
| *Window length of 1 s* |  |  |  |
| SVC | 61.6±1.7 | 76.5±1.2 | 0.0013 |
| Gaussian NB | 82.7±2.5 | 88.1±3.6 | 0.0012 |
| RF | 91.2±1.8 | 80.9±6.8 | 0.0002 |
| *k*NN | 94.4±1.4 | 86.5±2.8 | 0.0001 |
| LR | 90.4±2 | 79±2.3 | 0.0001 |
| LDA | 89.4±1.7 | 85.3±1.9 | 0.0001 |
| DT | 89.3±1.6 | 95.5±2.9 | 0.0001 |
| *Window length of 5 s* |  |  |  |
| SVC | 58.5±4.8 | 94.9±2.1 | 0.0001 |
| Gaussian NB | 82.6±3.7 | 95.8±1.3 | 0.0001 |
| RF | 88.1±4.7 | 96.9±2.1 | 0.0001 |
| *k*NN | 92.1±3.5 | 93.6±3.4 | 0.2374 |
| LR | 88.9±3.1 | 90.9±2.7 | 0.1495 |
| LDA | 86.8±6.1 | 93.3±2.9 | 0.0067 |
| DT | 84.7±7 | 98.2±1.3 | 0.0001 |
| *Window length of 10 s* |  |  |  |
| SVC | 60±9.3 | 94.4±1.5 | 0.0001 |
| Gaussian NB | 81.7±6.2 | 93.8±2.2 | 0.0001 |
| RF | 0.9±6.8 | 94.6±2.7 | 0.1423 |
| *k*NN | 88.8±4.4 | 99.3±0.4 | 0.0001 |
| LR | 82.1±4 | 95.6±1.3 | 0.0001 |
| LDA | 74.6±9.9 | 93.5±1.6 | 0.0001 |
| DT | 83.8±7.7 | 93.5±1.7 | 0.0011 |
| *Window length of 15 s* |  |  |  |
| SVC | 65.6±12.2 | 76.8±1.2 | 0.0099 |
| Gaussian NB | 86.3±9.7 | 84.1±0.7 | 0.496 |
| RF | 90±7.9 | 93.2±0.8 | 0.2158 |
| *k*NN | 80.6±9.1 | 98.1±0.3 | 0.0001 |
| LR | 88.8±9.2 | 93.5±0.9 | 0.1254 |
| LDA | 72.5±13.6 | 91.5±0.8 | 0.0003 |
| DT | 80±12.7 | 91.7±0.6 | 0.0098 |

**Supplementary Table 4.** Comparison of the accuracies (mean ± standard deviation) calculated from training and testing phases considering the different time window lengths using 30% of the extracted features.
