# Supplementary material for: Hand Resting Tremor Assessment of Healthy and Patients With Parkinson’s Disease: An Exploratory Machine Learning Study
Source: Front Bioeng Biotechnol. 2020 Jul 14;8:778. doi: 10.3389/fbioe.2020.00778 (PMC7381229; doi:10.3389/fbioe.2020.00778)
Supplement: Supplementary file 5 [file Table_5.DOCX]

| **Classifiers** | **Training phase** | **Testing phase** | **p-value** |
| --- | --- | --- | --- |
| *Window length of 1 s* |  |  |  |
| SVC | 62.7±2.1 | 85.1±1.1 | 0.0001 |
| Gaussian NB | 87.5±2.7 | 88.7±0.8 | 0.2142 |
| RF | 90.5±2.1 | 93.1±0.8 | 0.0029 |
| *k*NN | 90.8±1.7 | 94.2±0.6 | 0.0001 |
| LR | 88.9±2 | 91.1±1.1 | 0.0072 |
| LDA | 86.8±2.1 | 88.6±1.2 | 0.0337 |
| DT | 86.8±1.6 | 91.4±1.1 | 0.0001 |
| *Window length of 5 s* |  |  |  |
| SVC | 62.6±4.9 | 92.4±1.7 | 0.0001 |
| Gaussian NB | 83.8±4.2 | 88±2.4 | 0.0127 |
| RF | 86.6±4.1 | 94.6±1.5 | 0.0001 |
| *k*NN | 85.5±4.6 | 98.3±0.7 | 0.0001 |
| LR | 85.1±4.7 | 92.8±1.6 | 0.0005 |
| LDA | 85.3±5.4 | 91.4±2.2 | 0.0067 |
| DT | 82.1±5.7 | 92.7±1.4 | 0.0002 |
| *Window length of 10 s* |  |  |  |
| SVC | 62.9±9.9 | 91.8±3.9 | 0.0001 |
| Gaussian NB | 83.8±9.1 | 89.8±2.4 | 0.0676 |
| RF | 86.3±8.1 | 94.4±1.8 | 0.0117 |
| *k*NN | 88.8±5.9 | 96.2±1.3 | 0.003 |
| LR | 82.1±6.2 | 92±1.9 | 0.0006 |
| LDA | 81.7±5.6 | 91.7±1.9 | 0.0002 |
| DT | 78.8±6.4 | 91.8±2.4 | 0.0001 |
| *Window length of 15 s* |  |  |  |
| SVC | 70±13.4 | 91.5±7.3 | 0.0003 |
| Gaussian NB | 91.3±9.9 | 90.4±3.4 | 0.7977 |
| RF | 89.4±6.6 | 95.8±2.2 | 0.0146 |
| *k*NN | 80.6±5.4 | 94.7±2.3 | 0.0001 |
| LR | 91.3±5.3 | 91.7±2.5 | 0.825 |
| LDA | 78.8±7.9 | 89.9±3.2 | 0.0014 |
| DT | 83.6±9.9 | 92.1±4 | 0.0298 |

**Supplementary Table 5.** Comparison of the accuracies (mean ± standard deviation) calculated from training and testing phases considering the different time window lengths using 10% of the extracted features.
